# An attempt to construct a 7-item short version of the temperament and character inventory to predict the treatment response of patients with depression; a validation study

**DOI:** 10.1186/s12888-016-0997-0

**Published:** 2016-08-12

**Authors:** Tetsu Tomita, Norio Yasui-Furukori, Ayako Kaneda, Masamichi Ishioka, Norio Sugawara, Taku Nakagami, Kazuhiko Nakamura

**Affiliations:** 1 Department of Neuropsychiatry, Graduate School of Medicine, Hirosaki University, Hirosaki, 036-8562 Japan; 2 Department of Neuropsychiatry, Hirosaki-Aiseikai Hospital, Hirosaki, Japan; 3 Aomori Prefectural Center for Mental Health and Welfare, Aomori, Japan; 4 Department of Neuropsychiatry, Odate Municipal General Hospital, Odate, Japan

**Keywords:** Depression, Major depressive disorder, Response, Predict, Temperament and character inventory, Paroxetine, Antidepressants

## Abstract

**Background:**

The Temperament and Character Inventory (TCI) is a psychological test that is frequently used to assess personality traits. Many studies have shown the potential of the inventory to predict the treatment response of patients with major depressive disorder (MDD). Previously, we showed the association between 10 items of the TCI and the treatment response. In the present study, we reanalyzed the 10 items and aimed to provide cut-off values.

**Methods:**

This work is a secondary analysis of previously published work. Seventy-three patients were enrolled in the previously done study. Participants were treated with 10–40 mg/day of paroxetine for six weeks, and then the participants completed the TCI. The Montgomery-Asberg Depression Rating Scale (MADRS) was used to evaluate depression. The participants were divided into two groups (responders and non-responders). Using chi-squared tests, we reanalyzed the 10 items that had the strongest association with the treatment response in the previous study. We rated the answers to each item associated with the treatment response as a “1”, and the answers associated with a non-response were rated as a “0”. We calculated predictive scores using 10 models. Each model consisted of 1–10 scores of the best 1–10 items. We defined cut-off values for predicting treatment responses using a receiver operating characteristic (ROC) curve analysis.

**Results:**

Ranked by the strength of the association with the treatment response, items 174, 137, 70, 237, 106, 191, 34, 232, 161, and 215 of the TCI significantly predicted treatment responses. All predictive scores from models 1 to 10 significantly predicted treatment responses. The predictive score threshold of model 7 was 3/4, with an area under the curve of 0.825, and this model showed the highest odds and likelihood ratios (19.3 and 8.86, respectively).

**Conclusions:**

We might predict the treatment response of patients with MDD using TCI predictive scoring, including items 174, 137, 70, 237, 106, 191, and 34 and a cut-off value of 3/4.

**Electronic supplementary material:**

The online version of this article (doi:10.1186/s12888-016-0997-0) contains supplementary material, which is available to authorized users.

## Background

The Temperament and Character Inventory (TCI) is frequently used to assess personality traits [1]. The results of this questionnaire are summarized in seven dimensions; four dimensions are related to temperament (novelty seeking [NS], harm avoidance [HA], reward dependence [RD], and persistence [P]), and three dimensions are related to character (self-directedness [SD], cooperativeness [C], and self-transcendence [ST]). Many studies have shown an association between these TCI dimension values and disorders or certain mental conditions. Mitsui et al. investigated the association between suicide and the tendency of the TCI of university students and showed higher HA scores in subjects who committed suicide [2]. In another study, high NS and HA scores were reported to be risk factors for affective disorders [3].

Some studies reported the negative influence of personality disorders or personality dysfunction on the outcome of the treatment for the patients with depression. There were the association between comorbid or pre-existing personality disorders or personality dysfunction and poor response to treatment for depression [4, 5]. Additionally, the relationships between major depressive disorder (MDD) or depression and personality traits evaluated using TCI scores have been examined in many previous studies. High HA and low SD have been associated with depressed mood [6–14]. Furthermore, many studies have suggested that high HA scores are strongly associated with a depressive state or MDD on the TCI subscales [6, 9–14]. Therefore, MDD or a depressive state may be related to the personality traits that depend on higher HA.

Changes in TCI scores after treatment of patients with MDD were reported in previous studies [11, 15–18]. The results of these studies showed a significant decrease in HA scores after treatment or in the patients who showed a response to the treatment, and some of these studies reported changes in the SD, C, and ST scores [15–17]. In the study of Abrams et al., the inverse correlation between HA and improvement after treatment [18].

Thus, TCI dimensions are significantly correlated with MDD and depressive symptoms. Specific dimensions of the TCI may allow us to predict treatment responses in patients with MDD, which may prove beneficial in clinical settings. However, the TCI consists of 240 items, causing a potential burden to patients. We previously reported that some TCI items showed significant associations with paroxetine treatment responses in patients with MDD. It is also possible to predict treatment responses in patients with MDD using only 10 items (items 34, 70, 106, 137, 161, 174, 191, 215, 232 and 237) [19].

Our suggestion was novel, and predicting this response was difficult because we did not provide cut-off values with regard to how many answers associated with the responses that MDD patients had. In the present study, we reanalyzed the 10 items and aimed to provide these cut-off values for predicting treatment responses.

## Methods

### Participants

This work is a secondary analysis of previously published work.

Between December 2004 and September 2008, male and female patients between 19 and 72 years of age with a diagnosis of MDD, according to the Diagnostic and Statistical Manual (DSM)-IV, were recruited for participation in the previously done study. Participants were recruited from the Hospital of the Hirosaki University School of Medicine, Hirosaki-Aiseikai Hospital, Kuroishi-Akebono Hospital, and Odate Municipal General Hospital. Participants were included in the study if they scored higher than a 20 on the Montgomery Asberg Depression Rating Scale (MADRS) [20].

Of the 106 patients who were initially enrolled, 73 completed the study (25 males and 48 females). Thirty-three patients withdrew from the study, with 17 failing to complete the TCI questionnaire and 16 experiencing severe side effects from the medication. One patient included in the number of patients who failed to complete the TCI experienced a manic phase, and her diagnosis was changed to bipolar disorder. The mean ± standard deviation ages of completers, responders of completers, non-responders of completers, patients who withdrew because of failing to complete the TCI questionnaire, and patients who withdrew because of severe side effects were 45.6 ± 14.1 years, 46.9 ± 13.1 years, 44.1 ± 15.4 years, 50.7 ± 14.6 years, and 44.3 ± 17.1 years, respectively.

Approval was obtained from the Ethics Committee of the Hirosaki University School of Medicine prior to the study. The participants provided written informed consent after receiving a full description of the study.

### Measures

The MADRS consists of 10 items that are scored from 0 to 6. We excluded patients who had taken medications, including psychotropic agents, within the month prior to the start of the study. We also excluded individuals with clinically significant abnormal laboratory or electrocardiographic findings, histories of mental illness other than depression (e.g., bipolar disorder, mania, schizophrenia, epilepsy, alcohol/drug abuse, personality disorder), or clinically significant organic or neurological diseases.

The Udvalg for Kliniske Undersogelser (UKU) is a comprehensive scale that assesses the side effects of psychotropic drugs and consists of 48 items rated from 0 to 3 according to the presence or severity of the side effects [21]. A UKU score of 1 was defined as mild side effects, a UKU score of 2 was defined as moderate side effects, and a UKU of 3 was defined as severe side effects.

The 240-item Japanese version of the TCI was used at the beginning of the study. The TCI consisted of yes-no questionnaires and the 7 dimensions described above. The reliability and validity of this instrument had been previously established [22]. Kijima et al. reported the internal consistency of TCI among Japanese subjects as 0.64–0.85 [23].

### Protocol

A dose of 20 mg/day of paroxetine (Paxil, GlaxoSmithKline, Tokyo, Japan) was administered at 8 PM each day during the first week; the dose was then increased to 40 mg/day and administered from the second week to the sixth week. Blood samples were collected during treatment weeks 1, 2, and 6. Clinical symptoms were evaluated using the MADRS and the UKU side effect rating scale during treatment weeks 0, 1, 2, 4, and 6. If mild side effects (a UKU score of 1) were observed, the dose of paroxetine was maintained [21]. The dose was decreased if moderate side effects were observed (a UKU score of 2), and paroxetine administration was discontinued if severe side effects were observed (a UKU score of 3). The only other drugs administered during the study were diazepam (2–5 mg/day) for anxiety, brotizolam (0.25 mg/day) for insomnia, and sennoside (12–48 mg/day) for constipation.

### Data analysis and statistics

We defined responders as patients with MADRS improvements of > 50 % from baseline at week 6. A t-test and chi-square test were performed to compare demographic data and MADRS scores between the responders and non-responders.

In our previous study, we performed a chi-square test to evaluate the association between the results of the questionnaire and the responder rate for each of the 240 TCI items. We then identified the 10 items with the strongest associations with treatment response (items 34, 70, 106, 137, 161, 174, 191, 215, 232, and 237) [19]. In the present study, we reanalyzed the 10 items using a chi-square test. Answers with either a yes or no were associated with a non-response.

We rated the answers to each item associated with a response as a “1”, and those associated with a non-response were rated as a “0”. We constructed 10 models consisting of the TCI items. Each model consisted of scores from the 1 to 10 TCI items shown above. Models 1, 2, 3, 4, 5, 6, 7, 8, 9, and 10 consisted of scores from the items with the best, best and second, best to third, best to fourth, best to fifth, best to sixth, best to seventh, best to eighth, best to ninth, and best to tenth strongest association with treatment response, respectively. We defined the scores of each model as predictive scores. We compared the predictive scores between responders and non-responders using a t-test.

We used a receiver operating characteristic (ROC) curve to analyze the scores of each model to determine cut-off points using those that yielded the highest combined sensitivity and specificity for distinguishing responders and non-responders.

A *p*-value of < 0.05 was considered statistically significant. All analyses were performed using SPSS 22 for Windows (SPSS Japan Inc., Tokyo, Japan) and StatFlex version 6 (Artech Co, Ltd, Tokyo, Japan).

## Results

### Demographic and clinical characteristics

Table 1 shows the demographic and clinical characteristics of the patients, responders, and non-responders. The patients included 25 males and 48 females. The responders included 14 males and 28 females, and the non-responders included 11 males and 20 females. No significant difference in baseline MADRS score was found between the responders and non-responders. No significant differences in TCI dimensions were found, except for C.

**Table 1:** Demographic data and the comparison between responder and non-responders

	Responders (*n* = 42)	Non-responders (*n* = 31)	*p* value
Age	46.9 ± 13.1	44.1 ± 15.4	0.441
Sex (male:female)	14:28	11:20	0.522
Disease duration (months)	10.3 ± 17.0	16.6 ± 23.1	0.248
MADRS score			
0W	40.0 ± 8.6	39.2 ± 11.2	0.718
6W	5.7 ± 5.3	29.3 ± 9.5	0.000**
TCI dimensions			
NS	17.8 ± 4.1	16.6 ± 5.2	0.276
HA	27.0 ± 4.1	28.4 ± 3.9	0.136
RD	13.9 ± 3.6	13.7 ± 2.9	0.822
P	3.8 ± 1.7	4.3 ± 1.9	0.222
SD	21.2 ± 6.6	19.3 ± 6.0	0.199
C	27.8 ± 3.9	25.0 ± 5.7	0.026*
ST	11.8 ± 5.2	9.6 ± 4.8	0.065

*NS* novelty seeking, *HA* harm avoidance, *RD* reward dependence, *P* persistence, *SD* self-directedness, *C* cooperativeness, *ST* self-transcendence

*; *p* < 0.05, **; *p* < 0.01

### TCI items predicted the response and the predictive score models

The upper part of Table 2 shows the results of the chi-square tests to distinguish responders from non-responders. The TCI items are ordered by the strength of their relationship with treatment response. All 10 items showed significant differences. The lower part of Table 2 shows predictive score comparisons between responders and non-responders. All predictive scores were significant (Additional file 1).

**Table 2:** TCI items predicted the response and the predictive score models consisted of those TCI items

		Responders (*n* = 42)	Non-responders (*n* = 31)	*p* value
TCI items (yes:no)	174 (NS)	35:7	17:14	0.008**
	137 (C)	19:23	5:26	0.008**
	70^a^ (NS)	19:23	23:8	0.012*
	237^a^ (NS)	10:32	16:15	0.014*
	106^a^ (SD)	28:14	28:3	0.016*
	191 (NS)	19:23	6:25	0.019*
	34^a^ (NS)	11:31	16:15	0.024*
	232 (ST)	35:7	19:12	0.032*
	161 (C)	16:26	5:26	0.035*
	215 (ST)	14:28	4:27	0.040*
Predictive score	model 1	0.8 ± 0.4	0.5 ± 0.5	0.021*
	model 2	1.3 ± 0.7	0.7 ± 0.6	0.000**
	model 3	1.8 ± 0.9	1.0 ± 0.8	0.000**
	model 4	2.6 ± 1.0	1.5 ± 0.9	0.000**
	model 5	2.9 ± 1.3	1.5 ± 0.9	0.000**
	model 6	3.4 ± 1.4	1.7 ± 1.0	0.000**
	model 7	4.1 ± 1.6	2.2 ± 1.0	0.000**
	model 8	4.9 ± 1.7	2.8 ± 1.2	0.000**
	model 9	5.3 ± 1.7	3.0 ± 1.2	0.000**
	model 10	5.6 ± 1.9	3.1 ± 1.3	0.000**

*NS* novelty seeking, *SD* self-directedness, *C* cooperativeness, *ST* self-transcendence

^a^; “no” answers were associated with the response, *; *p* < 0.05, **; *p* < 0.01

### ROC curve analysis

**Fig. 1:**
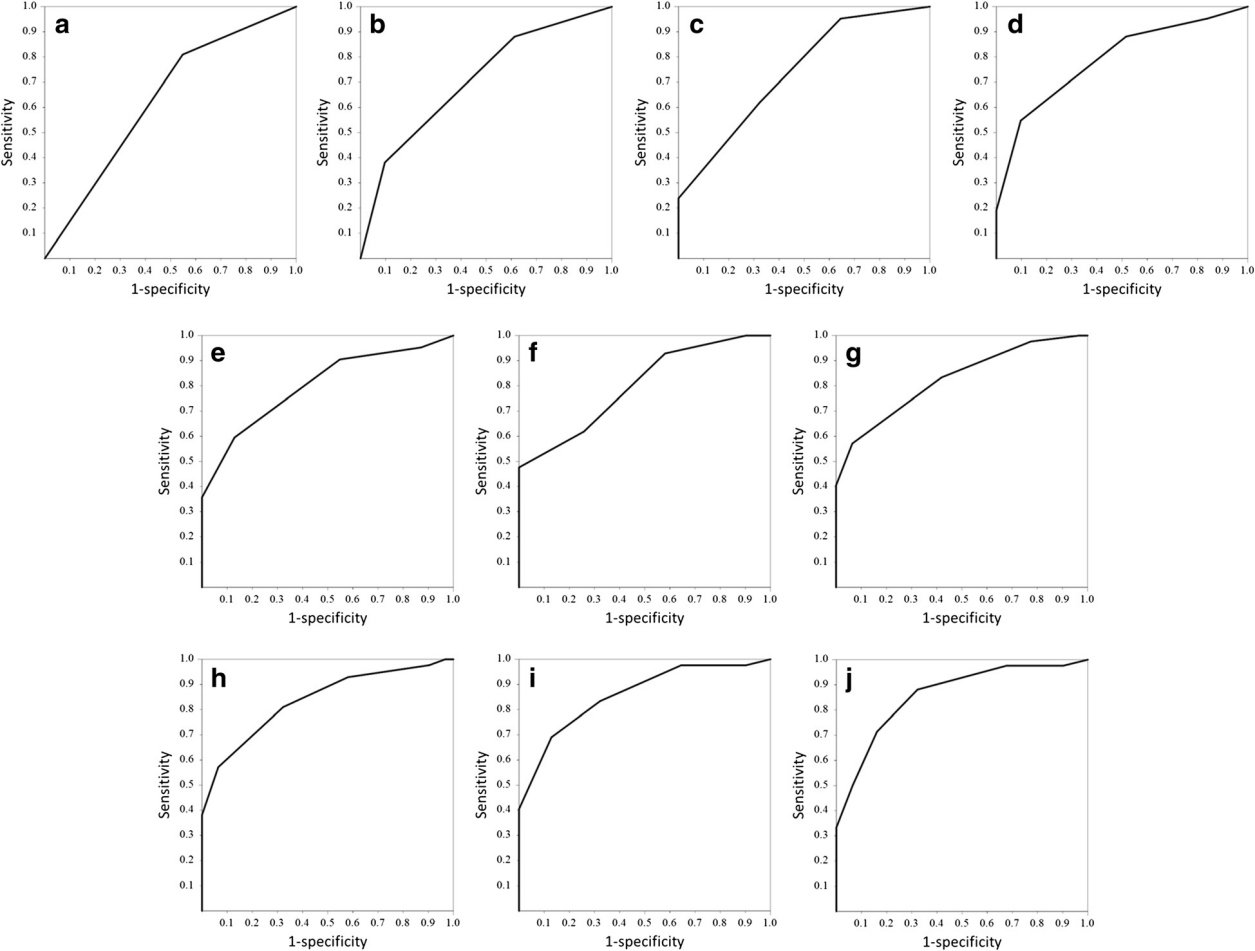
Receiver operating characteristic curves. **a-j** show the curves of model 1–10, respectively

Figure 1 shows the ROC curves for the predictive scores of the treatment response for models 1–10. The thresholds of the predictive scores of models 1–10 that gave maximal sensitivity and specificity for treatment response were 0/1, 0/1, 1/2, 2/3, 2/3, 2/3, 3/4, 3/4, 4/5 and 4/5. The sensitivity and specificity of the predictive scores for models 1–10 were 81.0 and 45.2 %; 88.1 and 38.7 %; 61.9 and 67.7 %; 54.8 and 90.3 %; 59.5 and 87.1 %; 61.9 and 74.2 %; 57.1 and 93.5 %; 81.0 and 67.7 %; 69.0 and 87.1 %; and 71.4 and 83.9 %. Table 3 shows the ROC curve analysis summary. The predictive score threshold of model 7 showed the highest odds and likelihood ratios (19.3 and 8.86, respectively).

**Table 3:** The results of receiver operating characteristic curve analysis

		Cut off	AUC	Sensitivity (%)	Specificity (%)	PPV (%)	NPV (%)	Odds ratio	Likelihood ratio	Accuracy (%)
model	1	0/1	0.631	81.0	45.2	66.7	63.6	3.5	1.48	65.8
	2	0/1	0.708	88.1	38.7	66.1	70.6	4.7	1.44	67.1
	3	1/2	0.738	61.9	67.7	72.2	56.8	3.4	1.92	64.4
	4	2/3	0.788	54.8	90.3	88.5	59.6	11.3	5.66	69.9
	5	2/3	0.801	59.5	87.1	86.2	61.4	9.9	4.61	71.2
	6	2/3	0.799	61.9	74.2	76.5	59.0	4.7	2.40	67.1
	7	3/4	0.825	57.1	93.5	92.3	61.7	19.3	8.86	72.6
	8	3/4	0.836	81.0	67.7	77.3	72.4	8.9	2.51	75.3
	9	4/5	0.858	69.0	87.1	87.9	67.5	15.1	5.35	76.7
	10	4/5	0.860	71.4	83.9	85.7	68.4	13.0	4.43	75.7

*AUC* area under curve, *PPV* positive predictive value, *NPV* positive predictive value

## Discussion

The present study demonstrated that the predictive scores of models including TCI items associated with treatment responses are able to predict treatment response. Cut-off values were also established that could distinguish responders from non-responders. This is the first study to utilize the TCI items associated with treatment responses that were reported previously [19].

In the present study, the areas under curve (AUC) of models 5, 7, 8, 9, and 10 were over 0.800, with the AUC of model 10 being the highest (0.860). However, the pairwise analysis to compare the AUCs of the abovementioned models demonstrated no significant differences. Model 7 showed the highest odds and likelihood ratios. To predict treatment responses in patients with MDD without overburdening patients, we should accept model 7 as the predictive model (including items 174, 137, 70, 237, 106, 191 and 34) and use a cut-off value of 3/4 for the predictive score.

Several previous studies have evaluated the ability of different instruments to predict treatment response in patients with MDD [24–26]. Kampman et al. reported that baseline and endpoint HA scores were correlated in patients with MDD using selective serotonin reuptake inhibitors (SSRIs) [24]. Furthermore, endpoint HA scores were shown to be associated with endpoint MADRS scores. A study using the Minnesota Multiphasic Personality Inventory-2 showed that the Social Introversion score was significantly associated with SSRI responses in patients with MDD [25]. In a study using the Five-Factor Model, Quilty et al. found that these personality traits were associated with treatment responses [26]. In the present and previous studies, we constructed a short version of the TCI to predict the treatment responses of patients with MDD using paroxetine. The short version of the TCI might be useful for predicting the treatment response of patients with MDD, but we need further study to investigate the personality traits of patients with MDD using the short version of the TCI in clinical settings.

Several previous studies have reported that HA and SD are closely related to depression and treatment responses in patients with MDD [6–14]. However, the independent variables that predicted treatment response according to logistic regression models 1–10 in the previous study included no HA items and just one item from the SD dimension [19]. The HA and SD scores were significantly associated with MDD. However, the use of different items within these dimensions may have counteracted the predictive effects of other items. In the present study, there were relatively higher dropout rate in the participants. The compliance of medication might be associated with character or temperament of the patients. The results of present study might reflect the characteristics of the specific patients who continued taking their medicines.

Ten TCI items were selected for inclusion in models 1–10 to calculate the predictive scores. The dimension containing the largest number of independent variables was NS. However, few studies have reported an association between the NS score and treatment response in patients with MDD. The finding that higher NS scores predict treatment response has already been discussed in a previous study [19]. Tome et al. reported that patients with MDD who had higher NS scores exhibited better treatment responses [27]. Genetic and biological studies related to NS scores have also been conducted. One study investigated a serotonin transporter promoter polymorphism (SERTPR) and reported a significant association between lower NS scores and homozygosity for the short allele [28]. A study investigating tryptophan hydroxylase (TPH1) genotypes and the TCI showed that the TPH1 A allele was associated with higher scores on the NS1 and NS2 subscales. Nakao et al. found an association between NS scores and the power of slow neuronal oscillations during the resting state using near-infrared spectroscopy [29]. Genetic or biological factors may influence the ability to predict treatment responses based on NS items.

None of the 10 items with the most significant associations with treatment response belonged to the HA dimension. The list of the 20 best items included only one HA item (Table 2) [19]. In contrast, the NS dimension contained the largest number of items in both the top 10 and top 20 lists. In a study using healthy student volunteers, Peirson et al. demonstrated that 5-HT2 receptor sensitivity was positively associated with HA, negatively associated with SD, and not correlated with NS [30]. The biological mechanism underlying the ability of NS to predict treatment response might be unrelated to serotonin receptor sensitivity. Some studies have reported associations between dopaminergic function and the NS dimension [31–33]. In a study using positron emission tomography, Suhara et al. demonstrated an association between NS scores and dopamine D2 receptor-binding potential [32]. Lee et al. found an association between NS and heterozygosity for the short allele of the dopamine D4 receptor gene in a female sample [33]. The ability of NS to significantly predict treatment responses in patients with MDD might involve a dopaminergic mechanism, and its association with the responders might not be associated with paroxetine or serotonergic functions.

The present study has some limitations. First, we excluded the patients with depression due to personality disorders or other disorders (e.g., bipolar disorder) and used only a single antidepressant, paroxetine, and did not evaluate the influence of additional drugs that were used, such as diazepam, brotizolam, and sennoside. The cut-off values might not apply to the patients with depression due to disorders except for MDD. According to Cloninger’s theory, some TCI dimensions are associated with neurotransmitters, including dopamine, serotonin, and noradrenaline [31]. Paroxetine and SSRIs mainly act on serotonergic mechanisms. Other classes of antidepressants act on other mechanisms, potentially changing the results. Other models or TCI items are needed to predict their effectiveness for patients with MDD. Further studies including other antidepressants would help to construct TCI predictive models for various treatment responses. Additional drugs might contribute to completion or responsivity to the treatment of the patients who used additional drugs. Second, the present study might have a selection bias. The results and discussion lack information on the 17 patients who did not complete the TCI. These participants' characters or temperaments might be different from those who completed the TCI. We should investigate whether the predictive model in the present study might predict the treatment responses of patients who did and did not complete the TCI. Third, TCI scores show only trait states of the patients with MDD, and we can evaluate the scores of items shown in the present study as important only by using TCI. TCI traits are only one of the factors associated with response to treatment. We cannot evaluate the important items shown in the present study by the impression of the patients in the clinical settings but we have to use TCI to evaluate the important items.

## Conclusions

We investigated and constructed a predictive model for MDD and treatment response that included TCI items associated with treatment response, and we determined the cut-off values of predictive scores. We might predict the treatment responses of patients with MDD using the predictive scores of items 174, 137, 70, 237, 106, 191 and 34, with a cut-off value of 3/4.

## Abbreviations

AUC, areas under curve; C, cooperativeness; DSM, diagnostic and statistical manual; HA, harm avoidance; MADRS, Montgomery Asberg Depression Rating Scale; MDD, major depressive disorder; NS, novelty seeking; P, persistence; RD, reward dependence; ROC, receiver operating characteristic; SD, self-directedness; SSRI, selective serotonin reuptake inhibitor; ST, self-transcendence; TCI, temperament and character inventory; TPH1, tryptophan hydroxylase; UKU, Udvalg for Kliniske Undersogelser

## Additional file

Additional file 1: Additional table. (DOCX 12 kb)

## References

[1] Cloninger CR, Svrakic DM, Przybeck TR (1993). A psychobiological model of temperament and character. Arch Gen Psychiatry.

[2] Mitsui N, Asakura S, Inoue T, Shimizu Y, Fujii Y, Kako Y, Tanaka T, Kitagawa N, Kusumi I (2013). Temperament and character profiles of Japanese university student suicide completers. Compr Psychiatry.

[3] Pawlak J, Dmitrzak-Weglarz M, Skibinska M, Szczepankiewicz A, Leszczynska-Rodziewicz A, Rajewska-Rager A, Maciukiewicz M, Czerski P, Hauser J (2013). Suicide attempts and psychological risk factors in patients with bipolar and unipolar affective disorder. Gen Hosp Psychiatry.

[4] Newton-Howes G, Tyrer P, Johnson T, Mulder R, Kool S, Dekker J, Schoevers R (2014). Influence of personality on the outcome of treatment in depression: systematic review and meta-analysis. J Pers Disord.

[5] Gorwood P, Rouillon F, Even C, Falissard B, Corruble E, Moran P (2010). Treatment response in major depression: effects of personality dysfunction and prior depression. Br J Psychiatry.

[6] Brown SL, Svrakic DM, Przybeck TR, Cloninger CR (1992). The relationship of personality to mood and anxiety states: a dimensional approach. J Psychiatr Res.

[7] Bayon C, Hill K, Svrakic DM, Przybeck TR, Cloninger CR (1996). Dimensional assessment of personality in an out-patient sample: relations of the systems of Millon and Cloninger. J Psychiatr Res.

[8] Cloninger CR, Bayon C, Svrakic DM (1998). Measurement of temperament and character in mood disorders: a model of fundamental states as personality types. J Affect Disord.

[9] Hansenne M, Reggers J, Pinto E, Kjiri K, Ajamier A, Ansseau M (1999). Temperament and character inventory (TCI) and depression. J Psychiatr Res.

[10] Naito M, Kijima N, Kitamura T (2000). Temperament and Character Inventory (TCI) as predictors of depression among Japanese college students. J Clin Psychol.

[11] Richter J, Eisemann M, Richter G (2000). Temperament and character during the course of unipolar depression among inpatients. Eur Arch Psychiatry Clin Neurosci.

[12] Farmer A, Mahmood A, Redman K, Harris T, Sadler S, McGuffin P (2003). A sib-pair study of the Temperament and Character Inventory scales in major depression. Arch Gen Psychiatry.

[13] Spittlehouse JK, Pearson JF, Luty SE, Mulder RT, Carter JD, McKenzie JM, Joyce PR (2010). Measures of temperament and character are differentially impacted on by depression severity. J Affect Disord.

[14] Kaneda A, Yasui-Furukori N, Nakagami T, Sato Y, Kaneko S (2011). The influence of personality factors on paroxetine response time in patients with major depression. J Affect Disord.

[15] Black KJ, Sheline YI (1997). Personality disorder scores improve with effective pharmacotherapy of depression. J Affect Disord.

[16] Corruble E, Duret C, Pelissolo A, Falissard B, Guelfi JD (2002). Early and delayed personality changes associated with depression recovery? A one-year follow-up study. Psychiatry Res.

[17] Hirano S, Sato T, Narita T, Kusunoki K, Ozaki N, Kimura S, Takahashi T, Sakado K, Uehara T (2002). Evaluating the state dependency of the Temperament and Character Inventory dimensions in patients with major depression: a methodological contribution. J Affect Disord.

[18] Abrams KY, Yune SK, Kim SJ, Jeon HJ, Han SJ, Hwang J, Sung YH, Lee KJ, Lyoo IK (2004). Trait and state aspects of harm avoidance and its implication for treatment in major depressive disorder, dysthymic disorder, and depressive personality disorder. Psychiatry Clin Neurosci.

[19] Tomita T, Ishioka M, Kaneda A, Sugawara N, Nakagami T, Nakamura K, Yasui-Furukori N (2014). An investigation of Temperament and Character Inventory items for predicting the response to paroxetine treatment in patients with major depressive disorder. J Affect Disord.

[20] Montgomery SA, Asberg M (1979). A new depression scale designed to be sensitive to change. Br J Psychiatry.

[21] Lingjaerde O, Ahlfors UG, Bech P, Dencker SJ, Elgen K (1987). The UKU side effect rating scale. A new comprehensive rating scale for psychotropic drugs and a cross-sectional study of side effects in neuroleptic-treated patients. Acta Psychiatr Scand Suppl.

[22] Kijima N, Saito R, Takeuchi M, Yoshino A, Ono Y, Kato M, Kitamura T (1996). Clininger’s seven-factor model of temperament and character and Japanese version of Temperament and Character Inventory (TCI). Archives of Psychiatric Diagnostics and Clinical Evaluation.

[23] Kijima N, Tanaka E, Suzuki N, Higuchi H, Kitamura T (2000). Reliability and validity of the Japanese version of the Temperament and Character Inventory. Psychol Rep.

[24] Kampman O, Poutanen O, Illi A, Setala-Soikkeli E, Viikki M, Nuolivirta T, Leinonen E (2012). Temperament profiles, major depression, and response to treatment with SSRIs in psychiatric outpatients. Eur Psychiatry.

[25] Kertzman S, Vainder M, Reznik I, Gotzlav Y, Weizman A, Kotler M, Iancu I (2012). Can Minnesota Multiphasic Personality Inventory-2 predict response to selective serotonin reuptake inhibitors in depressed outpatients?. Int Clin Psychopharmacol.

[26] Quilty LC, De Fruyt F, Rolland JP, Kennedy SH, Rouillon PF, Bagby RM (2008). Dimensional personality traits and treatment outcome in patients with major depressive disorder. J Affect Disord.

[27] Tome MB, Cloninger CR, Watson JP, Isaac MT (1997). Serotonergic autoreceptor blockade in the reduction of antidepressant latency: personality variables and response to paroxetine and pindolol. J Affect Disord.

[28] Serretti A, Mandelli L, Lorenzi C, Landoni S, Calati R, Insacco C, Cloninger CR (2006). Temperament and character in mood disorders: influence of DRD4, SERTPR, TPH and MAO-A polymorphisms. Neuropsychobiology.

[29] Nakao T, Matsumoto T, Shimizu D, Morita M, Yoshimura S, Northoff G, Morinobu S, Okamoto Y, Yamawaki S (2013). Resting state low-frequency fluctuations in prefrontal cortex reflect degrees of harm avoidance and novelty seeking: an exploratory NIRS study. Front in Systems Neuroscience.

[30] Peirson AR, Heuchert JW, Thomala L, Berk M, Plein H, Cloninger CR (1999). Relationship between serotonin and the temperament and character inventory. Psychiatry Res.

[31] Cloninger CR (1987). A systematic method for clinical description and classification of personality variants. A proposal. Arch Gen Psychiatry.

[32] Suhara T, Yasuno F, Sudo Y, Yamamoto M, Inoue M, Okubo Y, Suzuki K (2001). Dopamine D2 receptors in the insular cortex and the personality trait of novelty seeking. Neuroimage.

[33] Lee HJ, Lee HS, Kim YK, Kim L, Lee MS, Jung IK, Suh KY, Kim S (2003). D2 and D4 dopamine receptor gene polymorphisms and personality traits in a young Korean population. Am J Medical Genetics Part B, Neuropsychiatric Genetics.

