# Supplementary material for: An attempt to construct a 7-item short version of the temperament and character inventory to predict the treatment response of patients with depression; a validation study
Source: BMC Psychiatry. 2016 Aug 12;16:290. doi: 10.1186/s12888-016-0997-0 (PMC4983023; doi:10.1186/s12888-016-0997-0)
Supplement: Additional file 1: — Additional table. (DOCX 12 kb) [file 12888_2016_997_MOESM1_ESM.docx]

Additional file 1: Table S1

| Items |  | Subscale |  | Temperament and Character |
| --- | --- | --- | --- | --- |
| 174 |  | NS3 |  | Extravagance |
| 137 |  | C2 |  | Empathy |
| 70 |  | NS1 |  | Exploratory excitability |
| 237 |  | NS2 |  | Impulsiveness |
| 106 |  | SD3 |  | Resourcefulness |
| 191 |  | NS1 |  | Exploratory excitability |
| 34 |  | NS4 |  | Disorderliness |
| 232 |  | ST3 |  | Spiritual acceptance |
| 161 |  | C2 |  | Empathy |
| 215 |  | ST2 |  | Transpersonal identification |
| 174 |  | NS3 |  | Extravagance |

NS: novelty seeking, SD: self-directedness, C: cooperativeness, ST: self-transcendence
